# Possible role of ABO system in age-related diseases and longevity: a narrative review

**DOI:** 10.1186/1742-4933-11-16

**Published:** 2014-11-01

**Authors:** Claudia Rizzo, Calogero Caruso, Sonya Vasto

**Affiliations:** 1 Unit of Transfusion Medicine, University Hospital “Paolo Giaccone”, Palermo, Italy; 2 Department of Pathobiology and Medical and Forensic Biotechnologies, University of Palermo, Palermo, Italy; 3 National Center for Research, Institute of Biomedicine and Molecular Immunology, Palermo, Italy; 4 Department of Science and Biological, Chemical and Pharmaceutical Technologies, Institute of Biomedicine and Molecular Immunology, Palermo, Italy

**Keywords:** ABO, Cancer, Cardiovascular diseases, Inflammation, Longevity

## Abstract

ABO blood group antigens are expressed either on the surface of red blood cells either on a variety of other cells. Based on the available knowledge of the genes involved in their biosynthesis and their tissue distribution, their polymorphism has been suggested to provide intraspecies diversity allowing to cope with diverse and rapidly evolving pathogens. Accordingly, the different prevalence of ABO group genotypes among the populations has been demonstrated to be driven by malaria selection. In the similar manner, a particular ABO blood group may contribute to favour life-extension via biological mechanisms important for surviving or eluding serious disease. In this review, we will suggest the possible association of ABO group with age-related diseases and longevity taking into account the biological role of the ABO glycosyltransferases on some inflammatory mediators as adhesion molecules.

## Introduction

There are accumulating evidences that the ABO blood antigens might play a key role in various human diseases [1]. Historically, the ABO phenotype was one of the first marker involved in cancer susceptibility [2,3], whereas the first association between Human Leukocyte Antigens (HLA) and disease was described more recently: the association involved a cross-reactive group of HLA-B antigens and Hodgkin’s disease [4]. However, the identification of large numbers HLA-associated diseases counterpart our increased understanding of the genetic complexity of the HLA system and its extensive polymorphism [5], whereas data for ABO antigens are yet not so clear. The ABO molecules represent a complex membrane antigens widely expressed both on the surface of red blood cells (RBC) and many other cells extending the importance and the clinical significance of the ABO system beyond the transfusion medicine [6,7].

### The ABO system

The ABO antigen system occurs as a result of complex carbohydrate polymorphism mosaic of glycoproteins and glycolipids expressed on the surface of cells, or present in secretions, as glycan units [6,7].

The A and B antigens are inherited as Mendelian characteristics in a co-dominant autosomal fashion. These antigens are the enzymatic products of enzymes called glycosyltransferases that in turn synthesize the oligosaccharide epitopes. Thus, the A and B antigens are made by A and B glycosyltransferase, respectively (A-synthesizing 3-α-N-acetylgalactosaminyltransferase and the B-synthesizing 3-α-N-galactosaminyltransferase) while the O allele does not encode for a functional transferase. In this last case, the acceptor substrate, (H antigen: Fuc alpha1- > 2 Gal-) remains without a further modification and the A and B determinants are absent on surfaces of cells [6-8].

The A and B glycosyltransferases are type II membrane proteins located in the Golgi compartment, although soluble forms are found in plasma and other body fluids. The enzyme consists of a short transmembrane domain, a stem region and a catalytic domain that extends into the Golgi lumen. So, the A and B antigen synthesis occurs during normal glycosylation of proteins and lipids in the Golgi compartment. The precursor H substance is synthesized by one of two fucosyltransferases depending on the acceptor substrate used. The FUT1 gene that encodes the 2-α-fucosyltransferase (α2FucT1) is mainly responsible for the synthesis of the H antigen on carbohydrate precursors found on RBC. The closely related FUT2 gene encodes a very similar 2-α-fucosyltransferase (α2FucT2) that is expressed in epithelial cells [7,8].

As regards the organization of the ABO locus, the gene consists of seven coding exons and it is extended on 18 kb of genomic DNA. The sizes of the exons are included in a range of 28–688 bp, while the two major exons, 6 and 7, have a size of 1062 bp and contain most of the coding sequence. The single nucleotide deletion, found in a large number, but not all, of O alleles and responsible for the loss of the activity of the enzyme, is located in exon 6. The first of the seventh nucleotide substitutions, which distinguish the A, and B transferases, resides in coding exon 6. Exon 7 contains six nucleotide substitutions, resulting in four amino acid substitutions, that differentiate the A and B transferases [7,8].

ABO subgroups are distinguished by decreased amounts of antigens on RBC, so A2 subgroup RBC has less antigens than A1 subgroup (and so on for the other A and B subgroups). It is important to point out that the amount of antigens depends on glycosyltransferase activity [9].

The ABH sugars are found on lipids (approximately 10%) and proteins (approximately 90%) on the RBC surface as well as on many different tissues and cell types, including epithelial cells that line the lumen of the gastrointestinal, respiratory, and reproductive tracts as well as in salivary glands and skin. This wide distribution is a common feature for many of the carbohydrate blood groups, which has resulted in the term histo-blood group often being used to reflect this wide distribution. Accordingly, it has been suggested that these antigens, as the other glycoconjugates, are important mediators of intercellular adhesion and membrane signalling [7,8,10].

### Ageing and longevity

The ageing and longevity processes are determined by different genetic, epigenetic, stochastic and environmental factors. Among others, evidences from epidemiological studies on family of twins and long-lived individuals suggest a strong role for genetics. However, if genetics covers the 25% of the overall variation in human life span, another 25% is dependent on socio-economic factors in childhood, while adulthood and old age, including the socio-economic status and medical care, events may represent the remaining 50%. In the Western world, the development of the social-economics conditions, as medical care and quality of life, are responsible of a general improvement of the health population status with a consequent reduction of the overall morbidity and mortality, resulting in an overall increase of life expectancy [11,12].

Ageing can be defined as a decline in performance and fitness with advancing age, creating difficulty in adapting to new environmental situations. The post-maturational ageing process, in fact, is characterized by a diminished homeostasis and vulnerability of the body, responsible for a reduced response to environmental stimuli and stresses that lead to increased susceptibility and vulnerability to disease. Therefore, the mortality from all causes exponentially increases with age. In Western countries, the mortality rate increases in people over the age of 65, when compared with individuals between 25 and 44 years, because of heart disease (92 times), cancer (43 times), stroke (more than 100 times), chronic lung disease (greater than 100 times) and pneumonia and influenza (89 times), pointing out the role of the control of maintenance/repair systems as those involved in oxidative stress and immune-inflammatory responses, in the attainment of successful ageing [13-16].

Ageing is unnatural and damage is a fact of life. Ageing is not a programmed route but is a stochastic process resulting from accumulation of somatic damage implying a systemic loss of molecular fidelity. In ageing, the limited investments in maintenance and repair that are evolutionarily selected to assure reproduction and parental care brought to a minimal control of inflammation and oxidative stress. So, the ageing process is mostly stochastic, whereas the genome plays a role in determining longevity, which is regulated by the level of functional reserve reached at the time of reproductive age through natural selection. In other words, the effect of the duration of life is incidental because the main effect of the genome is to govern the events that occur until reproductive maturity [16-18].

Accordingly, demographic evidences suggest that longevity may be attained by different combinations of genes and environment with quantitative and qualitative differences in the different geographical areas where the population-specific genetic factors play a role in the longevity phenotype [19,20].

In this context, the study of centenarian offspring, a group of healthy elderly people with a familiar history of longevity, has helped gerontologists to better identify the correlation between genetic profile and hope of a healthy ageing. Previous studies have reported that centenarian offspring, like their centenarian parents, have genetic and immune system advantages, which reflect a minor risk to develop major age-related diseases, such as cardiovascular diseases (CVD), hypertension or diabetes mellitus as well as cancer [21,22].

Therefore, as previously stated, it is possible to assume that longevity may be influenced by polymorphisms of genes that control the immune-inflammatory responses as well as genes that modulate cardiovascular disease and cancer [20,23-25].

Different studies performed on mice have suggested that the Major Histocompatibility Complex (MHC), known to control a variety of immune functions, is associated with the life span of the strains. Hence, several studies have been performed in humans by analysing loci that regulate the immune response, as human MHC (HLA) and killer cell immunoglobulin-like receptor (KIR) genes regulating the cytolytic activity of natural killer cells. On the whole, the results suggest that HLA/KIR/longevity associations are population specific, being heavily affected by the population-specific genetic and environmental history [26-29].

In addition, as previously stated, alleles associated to CVD and cancer susceptibility have been shown to be not included in the genetic background favouring longevity, at least in some population, depending on the environmental stimuli [20,23].

### ABO and age related diseases

Over the time, several studies have tried a possible association between ABO groups and diseases although with contrasting results [1,9,30,31]. Many of the performed studies present serious challenges because of a number of confounding factors [32]: i) the incorrect sample size leading to selection bias and false positive associations (i.e. the lack of statistical power); ii) different inclusion criteria, unsuitable mixing of data (cohort effects) referred to people of different age, incorrect control matching, i.e. lack of selection from the same target population (stratification); iii) linkage disequilibrium, i.e. the associated ABO group may play no direct role, and the actual disease-predisposing polymorphism may be in linkage disequilibrium with the initially reported ABO association, which merely acts as a marker. Therefore, we will here report only the associations that have been confirmed relating to age-related diseases, cancer and CVD.

Cancer is generally recognized as an age-related disease [23,33]. In fact, incidence and mortality rates of most human cancers consistently increase with age up to 90 years, although they thereafter plateau and decline. The largest number of cancer cases occur in over 65 years in both sexes: the incidence of cancer is 12–36 times higher in subjects over 65 years compared to individuals aged between 25–44 years and 2–3 times more common than people aged between 45–64 years. The multistage model of carcinogenesis provides insight into the relationship between ageing and carcinogenesis, since ageing could be considered not so much as a determinant of cancer but as the condition, which results in a longer duration of exposure to carcinogens [23]. However, a low-grade systemic inflammation characterizes ageing and this pro-inflammatory status may underlie biological mechanisms responsible for age-related inflammatory diseases [14,24]. Clinical and epidemiological studies, in fact, show a strong association between chronic infections, inflammation and cancer and indicate that, even in tumours not directly linked to pathogens, the microenvironment is characterized by the presence of a smouldering inflammation, fuelled both by stromal leukocytes and senescent cells characteristic of ageing [23,24,34]. Therefore, the higher incidence of cancer in ageing could be due to pro-inflammatory state of ageing [23]. In any case, cancer and ageing are both fuelled by the accumulation of cellular damage, so they can simultaneously proceed [33].

Several studies have looked for the association between ABO blood group and cancer, however there are only evidences for pancreatic and gastric cancer [9,30,31].

The association with pancreatic cancer can be traced back to the 1960 study of Aird [35] who in 620 patients with pancreatic cancer found evidence of some strength that cancer of the pancreas is commoner in persons of group A than in persons of groups O or B. Following this pioneering study, in the following years many studies have been performed. As reviewed by Liumbruno and Franchini [9,30,31], cohort, case–control and meta-analysis studies clearly demonstrate the role of ABO blood group in pancreatic cancer. In particular, it is become clear the protective effect of O group and the association with A1 allele. The evidence that A1 allele, responsible for an increased glycosyltransferase activity, confers greater pancreatic cancer risk than A2 allele, focus on the biological role of glycans, as potential mechanism to explain the association, since glycoconjugates, such as ABO antigen, are important mediators of intercellular adhesion and membrane signalling, which are both critical to the progression and spread of malignant cells [10,36].

Concerning gastric cancer, also in this case, as stated in the Introduction, the pioneering study was performed by Aird [2], which in 1953 on 3,632 patients, highlighted a 20% increase of carcinoma of the stomach in group A as compared to group O. As discussed by Liumbruno e Franchini [9,30,31], most of the studies have confirmed that the risk of gastric cancer in blood group A is significantly higher than in non-A groups. Concerning the mechanisms, it has to point out that ABO blood group is a risk factor for progression towards gastric cancer in patients with Helicobacter pylori (Hp) infection, since in the gastric epithelium, the ABO blood groups antigens are one of the major functional receptor for Hp [36,37]. The association seems to be highly dependent on Hp cytotoxin associated gene (CagA) status, which is responsible for the secretion of the CagA virulence protein that, injected in the host cell cytosol, plays a relevant role in the precancerous lesion development. This might account for the lack of association in studies that did not take into account the prevalence of Hp infection in the population under study [36].

As regards age-related CVD, an important role is played by atherosclerotic disease, one of the main causes of mortality worldwide. Atherosclerosis is a chronic, progressive, multifactorial disease mostly affecting large and medium-sized elastic and muscular arteries: fatty-streak lesions are initiated by the accumulation of monocytes in subendothelial spaces, where they develop into lipid-laden macrophages, otherwise known as foam cells. Currently, multiple independent pathways of evidences suggest this pathological condition as a peculiar form of inflammation, triggered by cholesterol-rich lipoproteins and other noxious factors, such as cigarette smoke, diabetes mellitus and hypertension. Inflammation seems, indeed, to be the prevalent process of atherosclerosis, evocated by multiple risk factors and responsible for the altered arterial biology associated with atherosclerosis complications [20,38,39].

The initial stimulus inducing the inflammatory process has not yet been fully identified. However, endothelial dysfunction plays a crucial role in inflammation evocation. Several causes are associated with endothelial dysfunction, including the major number of traditional atherosclerosis risk factors, i.e. elevated low density lipoprotein values, free radicals caused by cigarettes smoking, hypertension, diabetes, elevated levels of homocysteine. Infections caused by Chlamydia pneumoniae, Herpes simplex virus, Cytomegalovirus and Hp also have been claimed to play a key factors in the disease. Thus, atherosclerosis may be considered as a characteristic response both to endothelium injury and to the consequent endothelial dysfunction. Endothelial dysfunction leads to compensatory responses modifying the normal homeostatic properties of the endothelium and favouring the expression of adhesion molecules, which, by binding to various classes of leukocytes, play a key role in the atherosclerotic process [20,38-41].

Complex interactions among the resident endothelial cells, the smooth muscle cells and the infiltrating monocytes and T lymphocytes determine the progression of fatty streaks into vascular lesions that block the normal blood flow and ultimately end into rupture of the atherosclerotic plaque, leading to either myocardial infarction or stroke [20,38,39].

However, the inflammatory process has a strong heritable component. Thus, the analysis of the genes that are key nodes of the inflammatory response might in part clarify the pathophysiology of atherosclerosis and its complications [14,20,24].

Several studies have documented the influence of ABO blood groups on plasma Willebrand factor (VWF) levels, hence of factor VIII plasma levels. ABH oligosaccaride structures have been identified on the N-linked oligosaccharide chains of VWF located in the A1 domain, which contains the binding site for platelet glycoprotein Ib. The VWF levels are approximately 25% higher in individuals who have a blood group other than O and it might depend on endothelial A, B glycosyltransferase enzymes, which generate A and B antigens, on the existing VWF “H” oligosaccharides. This addition to VWF might, in turn, influence its blood level [9,31,42-44].

On this basis, it is not surprising that a possible association between CVD and ABO blood group has been pointed out by several case–control, retrospective and meta-analysis studies. Although, most studies show an increased risk for CVD in subjects carrying non-O blood groups, there are conflicting results suggesting that the issue of the association between ABO blood group and CVD need further investigations [9,31]. On the other hand, large-scale genomic studies (GWAS) have revealed a central role for ABO antigens and blood levels of inflammatory mediators as soluble adhesion molecules, demonstrating that group O individuals show higher levels of inflammatory mediators. This might be due to glycosyltransferases activity, which might negatively influence shedding/cleavage of inflammatory molecules from the endothelium [9,20,31,45,46], see also below. In any case, this could explain the inconsistent results obtained.

### ABO and longevity

ABO antigens have been known for a long time and yet their biological meaning is still largely obscure [1,6]. Based on the available knowledge of the genes involved in their biosynthesis and their tissue distribution, their polymorphism has been suggested to provide intraspecies diversity allowing to cope with diverse and rapidly evolving pathogens [1,6]. Accordingly, the different prevalence of ABO group genotypes among the populations has been demonstrated to be driven by malaria selection [1,47,48]. In the similar manner, a particular ABO blood group may contribute to favour life-extension via biologica mechanisms important for surviving or eluding serious disease [6].

There are only five reports suggesting a possible association between ABO blood groups and ageing/longevity features, among those only two performed on centenarians and only one performed by molecular methods. In the first one, a significant increase of A blood type was observed in the healthy elderly male population over 64 years of age from UK [49], but it is not possible to consider this study for the very low age taken into account. In a study carried out on a small sample of very longevous Turkish population, no association was found [50]; however, the validity of age claims was very questionable because birth certificates were not available, so also this study cannot be considered.

A more recent study investigated the association between blood groups and life expectancy in Japanese population [51]. The authors compared frequencies of ABO blood groups in 269 centenarians living in Tokyo and those in 7153 regionally matched controls. Differences between centenarians and controls and between observed and expected frequencies were investigated by Chi Square tests. Group B was observed more frequently in centenarians than in controls, suggesting that group B might be associated with exceptional longevity. The authors suggested that group B individuals are more likely to survive age-related diseases rather than escape them, since 33% of the centenarians were free of age-related diseases, but this did not correlate with the group B.

In a further study, to validate these results, Brecher and Hay [52] collected data on the ABO blood groups of patients who died in a United States tertiary care hospital over a 1-year period. If group B was a marker for a longer lifespan, it would be expected that the percentage of group B patients would rise with age at the time of death and those of other blood groups would decline. A total of 772 patients were included in the study and data were presented as ABO proportion stratified by age. The authors found that the percentage of group B patients declined with age, and this result was statistically significant. None of the other blood groups showed a statistically significant increase or decrease when plotted against decade of death. Overall, these results suggest that group B is not a marker for longevity, at least in US.

We have recently investigated [53] the relationship between ABO group and longevity in a small sample of homogeneous Sicilian centenarians (n = 38) and young controls (n = 59). Our group of centenarians (age range 100–107) had no cardiac risk factors or other age-related diseases. The control group (age range 45–65) was recruited from blood donors and judged to be healthy on the basis of clinical history and blood tests (complete blood cell count, erythrocyte sedimentation rate, glucose, urea nitrogen, creatinine, electrolytes, C-reactive protein, liver function tests, iron, proteins, cholesterol and triglycerides). Samples were genotyped by molecular biology to determine ABO blood group and Chi Square analysis was used to determine the statistical significance of differences in ABO of centenarians and controls. Our pilot study shows a not-significant increase of A1 allele in Sicilian centenarians.

## Conclusions

As previously stated, blood groups seem to influence serum level of soluble adhesion molecules in blood stream. In particular, it is interesting to note that levels of serum soluble E-selectin, which represents an inflammatory marker of several diseases, including CVD, are higher in O/O individuals, whereas a single nucleotide polymorphism in A1 allele is associated with low levels of these inflammatory markers. A recent GWAS conducted on level of inflammatory markers in the Sardinian population highlighted the association between CVD and ABO locus and the association was established between this locus and interleukin(IL)-6 gene. Subjects homozygous for G allele in rs657152 SNP, corresponding to blood type O carriers, showed higher IL-6 circulating levels respect to non-O carriers, although the reason in unknown, reinforcing a relevant involvement of blood group antigens in inflammatory process. Indeed, previous studies demonstrated that a variant in ABO genes might explain the variation in soluble E-selectin levels [45,46,54-56].

As previously stated, several studies show that inflammatory gene variants, responsible for a low inflammatory response or a high anti-inflammatory response are associated with longevity, avoiding or delaying the onset of CVD [19-21]. In the generation of centenarians under study, the control of CVD, in fact plays a key role in the longevity attainment [19-21]. So, the Sicilian results, that need to be confirmed in a larger sample of centenarians, also taking into account the gender due to its relevance in immune-inflammatory responses [57], are in line with the previous statements. So, people carrying A1 allele should be advantaged in attaining longevity because of the lower levels of the serum soluble inflammatory marker E-selectin linked to this blood group, so avoiding or delaying cardiovascular events.

## Competing interests

The authors declare that they have no competing interests.

## Authors’ contributions

CR drafted the paper. All authors edited the paper and approved its final version.
